# Cinematic rendering improves the AO/OTA classification of distal femur fractures compared to volume rendering: a retrospective single-center study

**DOI:** 10.3389/fbioe.2023.1335759

**Published:** 2024-01-08

**Authors:** Song Chen, Xiong Wang, Zhenxin Zheng, Zhiqiang Fu

**Affiliations:** ^1^ Department of Orthopedics, The Quzhou Affiliated Hospital of Wenzhou Medical University, Quzhou People’s Hospital, Quzhou, Zhejiang, China; ^2^ Department of Orthopedics, Shanghai Baoshan Luodian Hospital, Shanghai, China

**Keywords:** distal femur fracture, AO/OTA classification, cinematic rendering, volume rendering, diagnostic confidence

## Abstract

**Purpose:** Correctly classifying distal femur fractures is essential for surgical treatment planning and patient prognosis. This study assesses the potential of Cinematic Rendering (CR) in classifying these fractures, emphasizing its reported ability to produce more realistic images than Volume Rendering (VR).

**Methods:** Data from 88 consecutive patients with distal femoral fractures collected between July 2013 and July 2020 were included. Two orthopedic surgeons independently evaluated the fractures using CR and VR. The inter-rater and intra-rater agreement was evaluated by using the Cicchetti-Allison weighted Kappa method. Accuracy, precision, recall, and F1 score were also calculated. Diagnostic confidence scores (DCSs) for both imaging methods were compared using chi-square or Fisher’s exact tests.

**Results:** CR reconstruction yielded excellent inter-observer (Kappa = 0.989) and intra-observer (Kappa = 0.992) agreement, outperforming VR (Kappa = 0.941 and 0.905, respectively). While metrics like accuracy, precision, recall, and F1 scores were higher for CR, the difference was not statistically significant (*p* > 0.05). However, DCAs significantly favored CR (*p* < 0.05).

**Conclusion:** CR offers a superior visualization of distal femur fractures than VR. It enhances fracture classification accuracy and bolsters diagnostic confidence. The high inter- and intra-observer agreement underscores its reliability, suggesting its potential clinical importance.

## Introduction

Accurate preoperative evaluation is vital in diagnosing and managing distal femoral fractures. Traditionally, anteroposterior, and lateral radiographs serve as primary tools. Yet, patient discomfort often leads to compromised imaging quality, affecting an orthopaedic surgeon’s diagnostic capability (Pinto et al., 2018; Grassi et al., 2023).

The advent of computed tomography (CT) offered enhanced diagnostic accuracy over conventional radiographs in fracture diagnostics (Krastman et al., 2020; Yang and Wang, 2021). However, interpretation of conventional two-dimensional (2D) CT images demands a fusion of anatomical knowledge and inference, making the process susceptible to variation based on the clinician’s experience.

Recently, three-dimensional (3D) imaging techniques, such as volume rendering (VR), have improved clinical outcomes in fracture management (Duran et al., 2019; Essa et al., 2022). Cinematic rendering (CR) is an emerging 3D imaging modality, differentiating itself from VR through a complex global illumination model, creating detailed, lifelike images (Chu et al., 2019; Al Khalifah et al., 2022; Recht et al., 2023). Preliminary studies have suggested superior shape perception and depth using CR over VR (Ebert et al., 2017; Eid et al., 2017; Johnson et al., 2017; Elshafei et al., 2019; Boven et al., 2020; Binder et al., 2021). Yet, its applicability in classifying distal femoral fractures, particularly based on the Arbeitsgemeinschaftfür Osteosynthesefragen Foundation and the Orthopaedic Trauma Association (AO/OTA) classification system, remains unexplored.

This study aims to assess the value of CR against VR in classifying distal femoral fractures using the AO/OTA system (Meinberg et al., 2018). We hypothesize that CR will elevate diagnostic accuracy, enhance intra- and interobserver agreement, and increase diagnostic confidence among orthopaedic surgeons.

### Key contributions of this study

• Assessment of the effectiveness of CR in classifying distal femoral fractures using the AO/OTA system.
• Comparison of CR with VR in terms of diagnostic accuracy, intra- and interobserver agreement, and diagnostic confidence.
• Exploration of CR’s potential to enhance fracture management in orthopaedic surgery.

### Overview of following sections

The subsequent sections of this paper will detail the evolution and current state of imaging techniques in fracture diagnosis, our methodology, the results of our comparative analysis, a discussion of these findings, and conclusions drawn from our study.

## Materials and methods

### Study population

Clinical and imaging data from 88 consecutive patients with distal femoral fractures, gathered between July 2013 to July 2020 at our hospital were retrospectively scrutinized. Inclusion criteria for the cases entailed: 1) preoperative diagnosis of distal femur fracture, confirmed intraoperatively; 2) age ≥18 years; 3) complete imaging data available. Exclusion criteria included poor imaging quality post-reconstruction. Following these criteria, two patients were excluded due to age, and four due to incomplete imaging.

Prior to evaluation, an experienced senior surgeon (Z.Q.) with 2 decades of expertise in treating distal femur fractures, in conjunction with VR and CR techniques, categorized all fractures per the AO/OTA system. This was formulated as the gold standard. All patient information was thoroughly anonymized before evaluation. A single resident (S.C.) collected and collated all patient information but did not participate in any assessment experiments.

This study adhered to the ethical principles outlined in the Declaration of Helsinki. It received approval from the institutional review board of our institution, with the approval number [2023CL171]. As this study was retrospective in nature, the requirement for patient informed consent was waived.

### Evaluation process

Before evaluation, the raw CT data for each patient were processed to obtain thin-layer reconstructions. The term “thin-layer” refers to the technique of reconstructing CT images with a reduced slice thickness, typically in the range of 0.625–1 mm. This approach enhances the resolution and detail of the images, providing a more precise representation of the bone structure and fracture details. These high-resolution thin-layer CT images were individually preserved in Digital Imaging and Communications in Medicine (DICOM) format within consecutively numbered folders for subsequent analysis. For each assessment, the order was randomized, prompting the generation of a random set of numbers, after which the folders were renamed and reordered. The VR assessments were conducted using the E-3D digital medical modelling and planning system (version 18.02), while the CR assessments were executed using Mevislab software (version 3.4.1).

Two orthopedic surgeons from different hospitals with a combined clinical experience of 10 years (observer 1, XW.) and 14 years (observer 2, ZZ.) independently conducted the assessments. Both surgeons underwent training until they were adept at utilizing both software for fracture rendering and reconstruction. They were also at liberty to remove irrelevant bone structures to avoid occlusion and thus clearly display the distal femur fracture. Both evaluated the fractures according to the AO/OTA classifying criteria and assigned a diagnostic confidence score (DCS) to each completed fracture typing. This score varied from 0 to 5, with 0 indicating no confidence in the typing results and 5 signifying supreme confidence in their diagnosis. Throughout the evaluation process, the two surgeons were instructed to refrain from communicating their findings until the entire evaluation process was finalized.

The entire evaluation process comprised four occasions. The initial assessment witnessed both surgeons independently evaluating using VR imaging. Six weeks later, a similar independent evaluation was conducted using CR imaging. Following another 6-week interval, evaluations were performed first by observer 1 and then using VR and CR imaging techniques. The interval between this round of evaluations remained at 6 weeks. The results of the first round of evaluations were utilized to calculate interobserver agreement, while the results of the second round of evaluations for observer 1 were used to compute intra-observer agreement. Accuracy, precision, recall, F1 score, and DCS for fracture classifying were analyzed from the data recorded in the first round.

### Statistical analysis

Cicchetti-Allison weighted Kappa values were utilized to measure inter-observer and intra-observer agreement (Vanbelle, 2017). The strength of agreement was ranked according to the criteria suggested by Landis and Koch (Landis and Koch, 1977). Kappa values ranging from 0.00 to 0.20 represented slight agreement; 0.21 to 0.40 represented fair agreement; 0.41 to 0.60 represented moderate agreement; 0.61 to 0.80 represented substantial agreement; and 0.81 to 1.0 represented almost perfect agreement.

Quantitative data that followed a normal distribution were presented as mean ± standard deviation (SD), while non-normally distributed data were expressed as median with interquartile range. Qualitative data were depicted as frequencies and percentages. Normality was assessed using the Shapiro-Wilk test, with a significance level set at 0.05. For this test, the null hypothesis was that the data follows a normal distribution, and the alternative hypothesis was that it does not.

Differences in DCS between the two assessors were evaluated using the independent *t*-test for normally distributed data, with the null hypothesis being no difference in means between groups, and the alternative hypothesis being a significant difference in means. If data were not normally distributed, the Mann-Whitney U test was applied, where the null hypothesis was that the distributions are equal, and the alternative hypothesis was that one distribution is stochastically greater than the other.

For comparing the accuracy of imaging techniques, either the chi-square test or Fisher’s exact test was employed, depending on the sample size and the expected cell frequencies, i.e., when more than 20% of cells have expected frequencies less than 5, the Fisher’s exact test was applied. Statistical significance was ascertained by a *p*-value below 0.05. The null hypothesis for these tests was that there is no difference in proportions between groups, while the alternative hypothesis was that there is a significant difference in proportions.

Precision, recall, and F1 scores were other metrics to assess the two imaging modalities. Precision represented the fraction of accurately identified distal femur fractures among all fractures classified as such by the evaluator. Recall measured the proportion of correctly pinpointed fractures by the examiner relative to the total actual fractures of that specific type. The F1 score served as the harmonic balance of precision and recall.

Analyses involving the Kappa, DCS, and accuracy metrics were executed in R software (version 4.1.0). Meanwhile, computations for precision, recall, and the F1 score were conducted using Python (version 3.8.8).

## Results

### Patient characteristics

**FIGURE 1:**
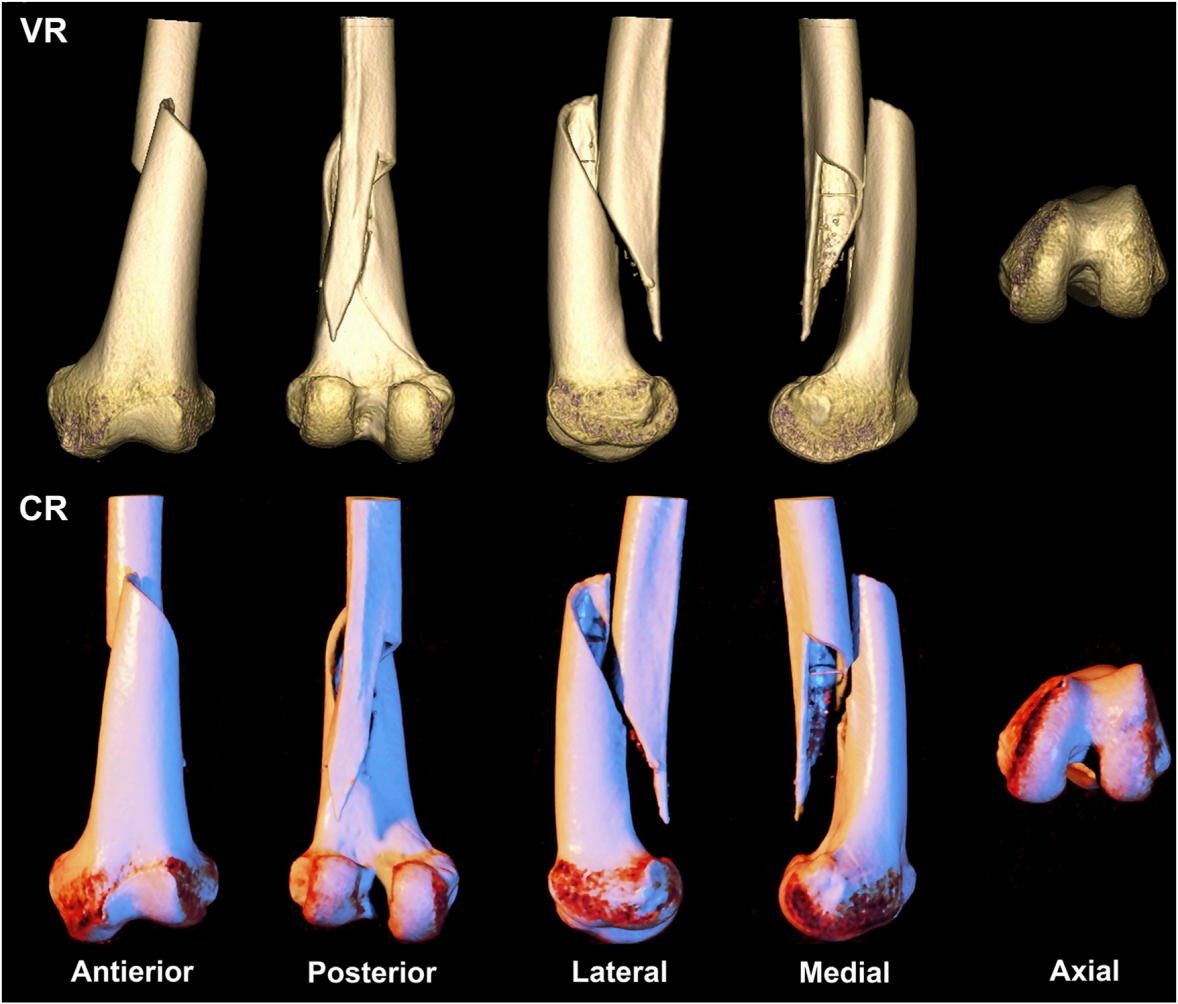
A 69-year-old woman presents with a straightforward fracture of the left femoral shaft and epiphysis, classified as type AO/OTA 33A1. The first row depicts a volumetric rendering reconstruction, while the second row showcases a cinematic rendering reconstruction.

**FIGURE 2:**
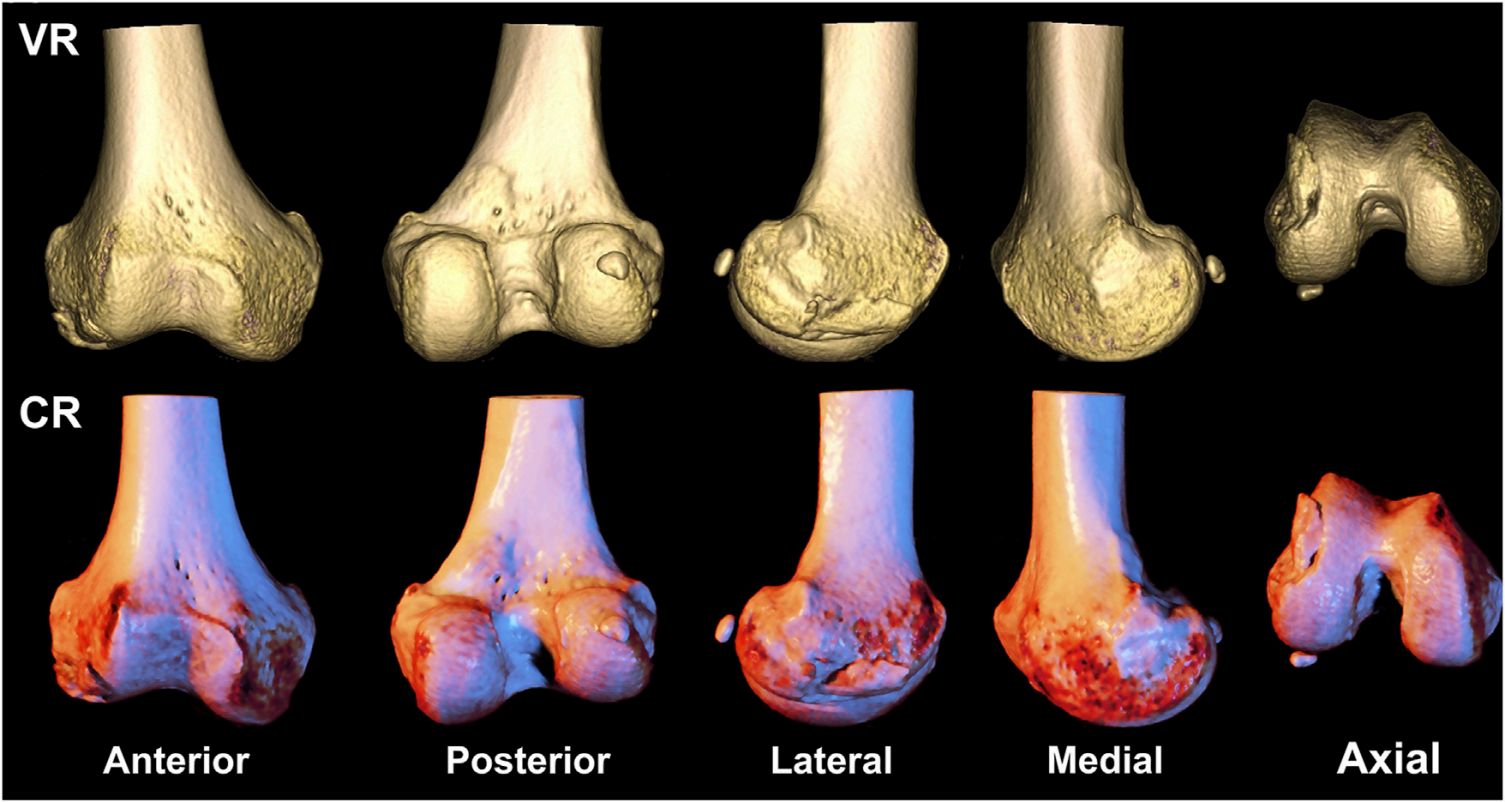
A 58-year-old gentleman presents with a fracture of the right distal femoral epicondyle, featuring fracture line involvement of the articular surface, classified as type AO/OTA 33B1. The first row depicts a volumetric rendering reconstruction, while the second row showcases a cinematic rendering reconstruction.

**FIGURE 3:**
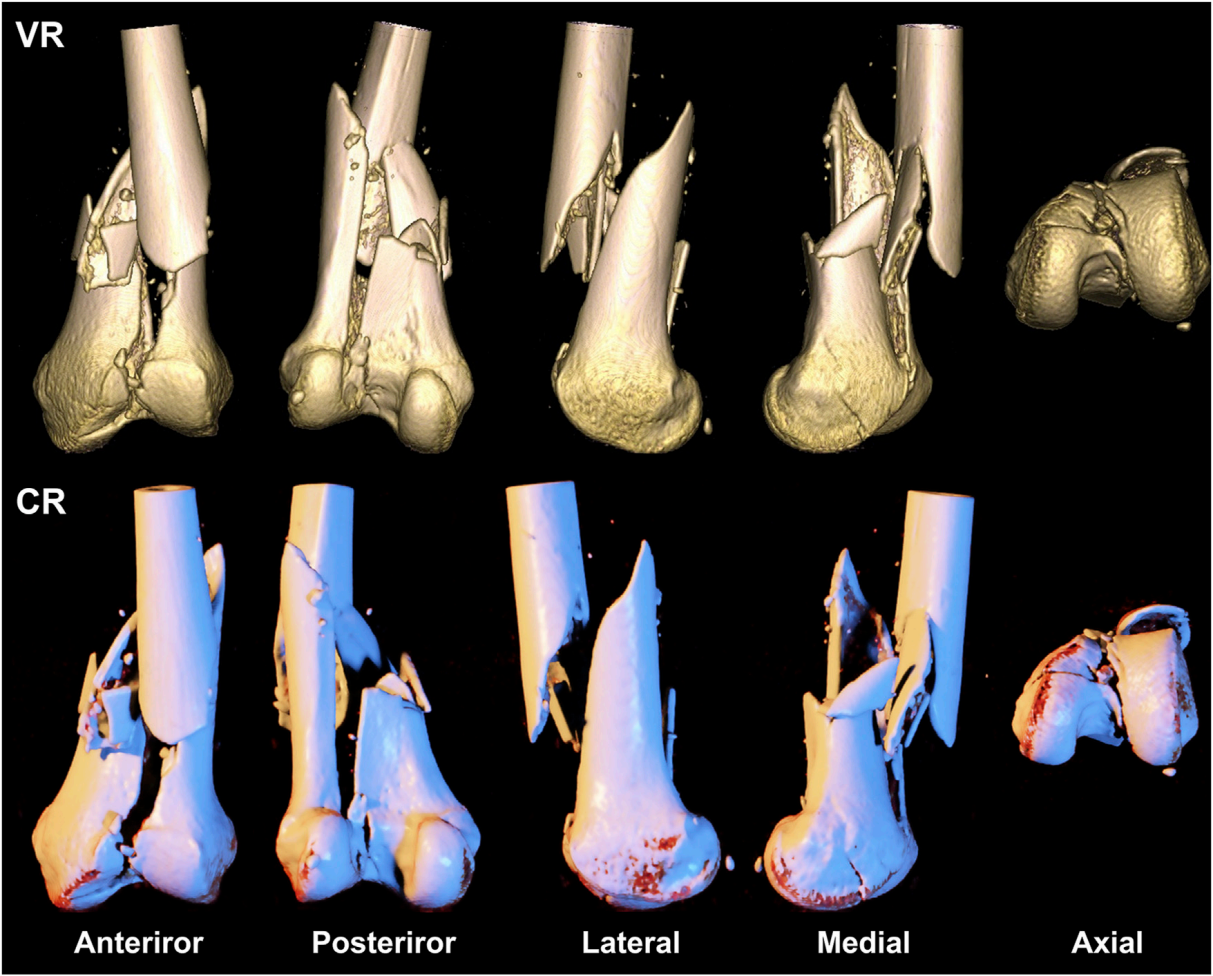
A 39-year-old woman presents with a comminuted fracture of the left distal femoral epiphysis and articular surface, classified as type AO/OTA 33C3. The first row depicts a volumetric rendering reconstruction, while the second row showcases a cinematic rendering reconstruction.

Among the 82 distal femoral fractures enrolled in the study, the age range was from 18 to 92 years, with a mean of 57.5 years, and the body mass index (BMI) was 23.8 ± 3.1 kg m^−2^. Males constituted 50.0% (41 patients) of the population, and 56.1% (46 patients) sustained injuries on the left side. AO/OTA type 33A fractures were found in 48 cases (58.5%), type 33B fractures in 13 cases (15.9%), and type 33C fractures in 21 cases (25.6%) (Figures 1–3, Supplementary Figures S1–S6). As for the causative events leading to the fractures in our cohort, the majority resulted from slips (48.8%, 40 patients), followed by motor vehicle accidents (28.0%, 23 patients), falls from height (14.6%, 12 patients), and crush injuries (8.5%, 7 patients) (Table 1).

**TABLE 1:** Patient demographics and fracture characteristics.

Characteristics	Values (n = 82)
Age (years), mean ± SD	57.5 ± 19.1
BMI (kg.m^-2^), mean ± SD	23.8 ± 3.1
Gender, no. (%)	
Male	41 (50.0)
Female	41 (50.0)
Injured side, no. (%)	
Left	46 (56.1)
Right	36 (43.9)
Injury mechanism, no. (%)	
MVA	23 (28.0)
Slip	40 (48.8)
Fall	12 (14.6)
Crushing	7 (8.5)
AO/OTA classification, no. (%)	
33A1	15 (18.3)
33A2	10 (12.2)
33A3	23 (28.0)
33B1	3 (3.7)
33B2	8 (9.8)
33B3	2 (2.4)
33C1	1 (1.2)
33C2	11 (13.4)
33C3	9 (11.0)

**Abbreviations:** AO/OTA, Arbeitsgemeinschaftfür Osteosynthesefragen Foundation and the Orthopaedic Trauma Association; BMI, body mass index; MVA, motor vehicle accident; SD, standard deviation.

### Inter- and intra-observer agreement

**FIGURE 4:**
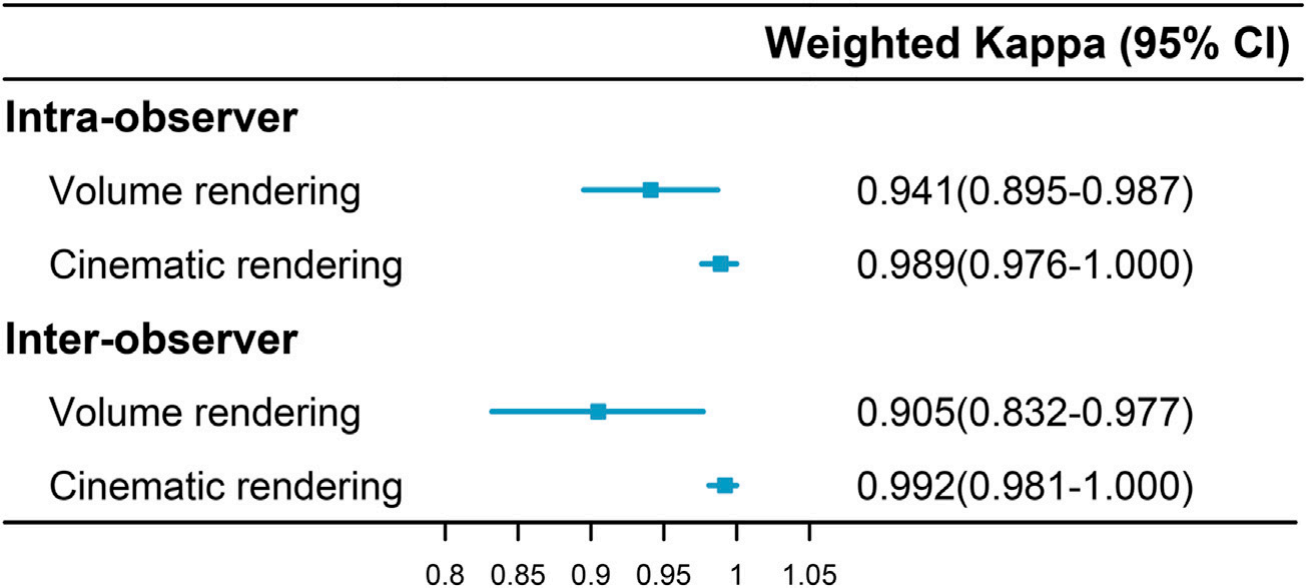
Intra- and inter-observer agreement in fracture classification via two imaging modalities.

VR and CR imaging of the fracture is shown in Figures 1–3. When fractures were typed using the VR imaging modality, the inter-observer weighted Kappa value was 0.905 (*p* < 0.05), with excellent agreement, and the intra-observer weighted Kappa value was 0.941 (*p* < 0.05), also with excellent agreement. When fractures were typed using the CR imaging modality, the interobserver-weighted Kappa value increased to 0.992 (*p* < 0.05), which graded as excellent agreement, and the intraobserver-weighted Kappa value also increased to 0.989 (*p* < 0.05), which also graded as excellent agreement (Figure 4).

### Metrics for classification results evaluation

**FIGURE 5:**
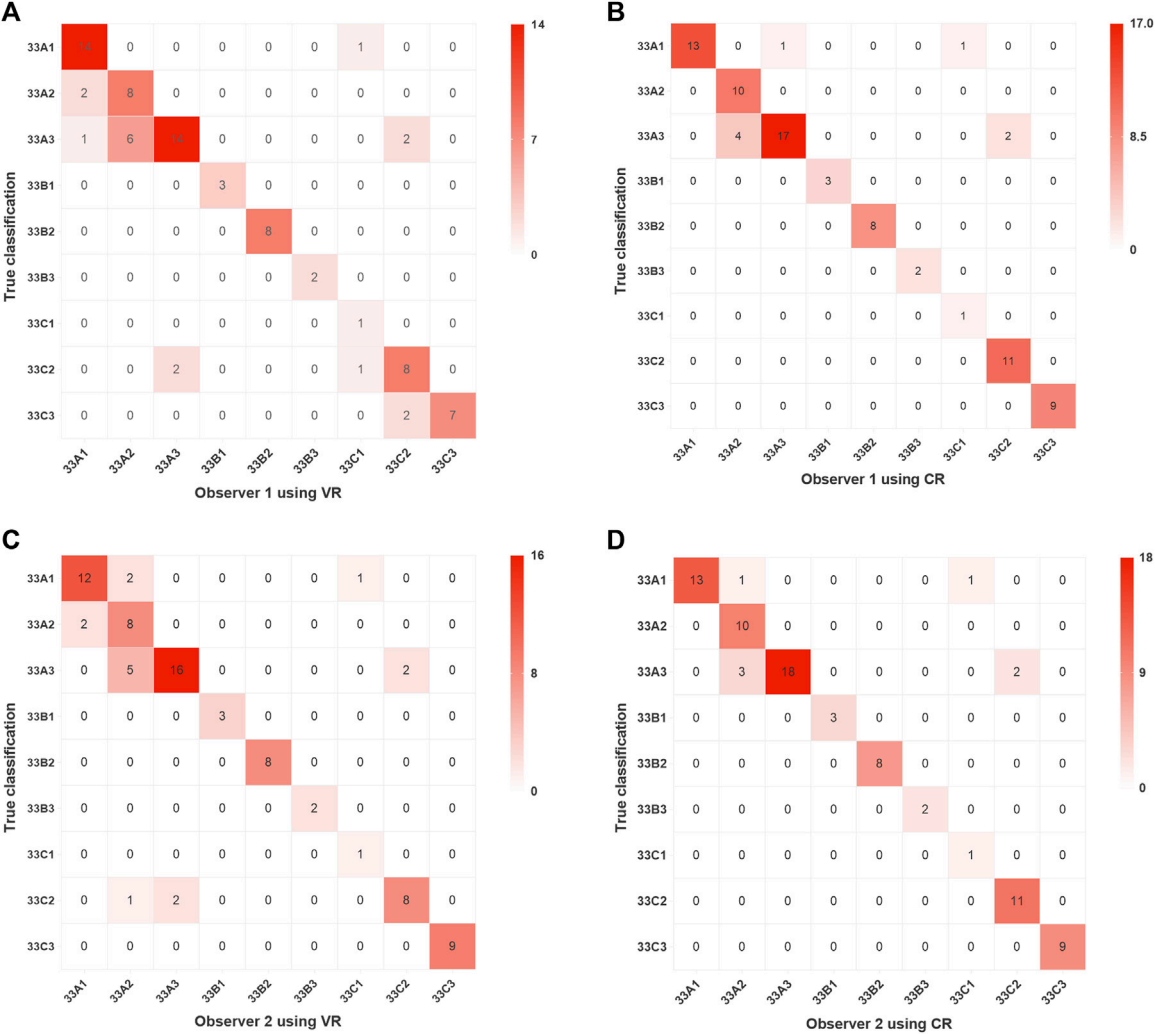
Confusion matrix visualizations of two assessors following classification of distal femoral fractures utilizing two imaging modalities. Assessment results of Observer 1 using VR and CR are presented in **(A,B)**, respectively, while those of Observer 2 using VR and CR are shown in **(C,D)**, respectively. VR, volumetric rendering; CR, cinematic rendering.

The results of classification using VR and CR imaging in observers 1 and 2 are detailed in Figure 5. Observer 1 achieved an accuracy of 0.79 with VR imaging and 0.90 with CR imaging, indicating a notable discrepancy of 11% between the two (95% Confidence interval (CI), 0.05%–21.95%; χ^2^ = 3.82; *p* = 0.051). Observer 2 demonstrated an accuracy of 0.82 using VR imaging and ascended to 0.92 upon employing CR imaging, thereby yielding a marginal difference of 10% (95% CI, −0.18%–20.18%; χ^2^ = 2.92; *p* = 0.088).

**FIGURE 6:**
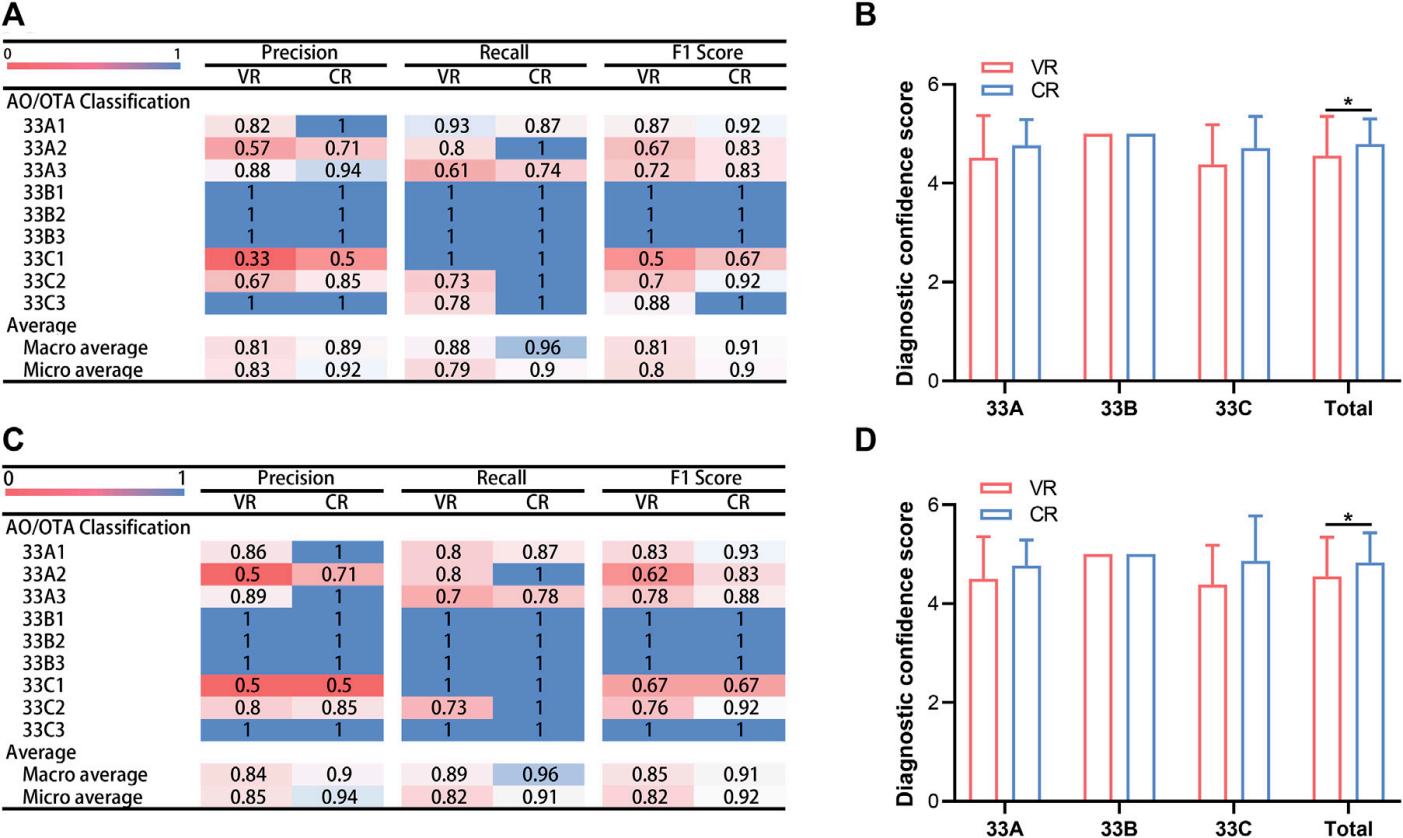
Comparative analysis of assessment outcomes and diagnostic confidence scores by two evaluators utilizing two imaging modalities. **(A)** Accuracy, recall and F1 score garnered by observer 1 across two distinct imaging modalities. **(B)** Diagnostic confidence scores for observer 1 using two different imaging modalities. **(C)** Accuracy, recall and F1 score garnered by observer 2 across two distinct imaging modalities. **(D)** Diagnostic confidence scores for observer 2 using two different imaging modalities. ^∗^ *p* < 0.05. VR, volumetric rendering; CR, cinematic rendering.

Precision for each subtype of the distal femur using VR imaging varied from 0.33 to 1.00 for observer 1 and from 0.50 to 1.00 with CR imaging. Concurrently, recall spanned from 0.61 to 1.00 and from 0.74 to 1.00, respectively, while F1 scores exhibited a range of 0.5–1.00 and 0.67 to 1.00, respectively, as evidenced in Figure 6A. For observer 2, precision oscillated between 0.50 and 1.00 using VR imaging and was congruent using CR imaging, with recall shifting between 0.61 to 1.00 and 0.74 to 1.00, respectively; and F1 scores fluctuating between 0.5 to 1.00 and 0.67 to 1.00, respectively, as depicted in Figure 6C.

Post-classifying, the VR and CR imaging techniques employed by observer 1 demonstrated macro-average and micro-average precisions of 0.81 and 0.89 and 0.83 and 0.92, respectively; macro-average and micro-average recalls of 0.88 and 0.96 and 0.79 and 0.90, respectively; and macro-average and micro-average F1 scores of 0.81 and 0.91 and 0.80 and 0.90, respectively, as portrayed in Figure 6A. Following fractionation with CR imaging, observer 2 obtained macro-average and micro-average precisions of 0.84 and 0.90 and 0.85 and 0.94, respectively; macro-average and micro-average recalls of 0.89 and 0.96 and 0.82 and 0.91, respectively; and macro-average and micro-average F1 scores of 0.85 and 0.91 and 0.82 and 0.92, respectively, as illustrated in Figure 6C.

Upon evaluating fractures via CR imaging, most values superseded those procured from VR imaging assessments. In observer 1’s evaluation of type 33A distal femur fractures, type 33A2 showed inferior accuracy and F1 scores compared to types 33A1 and 33A3, and type 33A3 presented the least recall. In the classification of type 33B fractures, precision, recall, and F1 scores uniformly peaked at 1. For type 33C fractures, type 33C1 displayed diminished precision and F1 scores compared to types 33C2 and 33C3, despite having the highest recalls. Observer 2 displayed analogous results, as encapsulated in Figures 6A,C.

### Comparison between DCSs

Figures 6B,D elucidate the DCSs attained by the two observers using VR and CR imaging techniques. The mean DCSs for observer 1, utilizing VR and CR imaging techniques, were 4.56 ± 0.79 and 4.79 ± 0.51, respectively, a disparity that was deemed statistically significant (*p* < 0.05). In the diagnosis of type 33A and 33C fractures, the mean DCSs for both imaging methods were 4.52 ± 0.85 and 4.77 ± 0.52, and 4.38 ± 0.80 and 4.71 ± 0.64, respectively (*p* > 0.05).

For observer 2, the mean DCSs yielded via VR and CR imaging techniques were 4.55 ± 0.79 and 4.83 ± 0.60, respectively. This variation was likewise significant (*p* < 0.05). When diagnosing type 33A and 33C fractures, the mean DCSs extracted from the two imaging techniques were 4.50 ± 0.85 and 4.77 ± 0.52, and 4.38 ± 0.80 and 4.86 ± 0.91, respectively (*p* > 0.05). However, in the diagnosis of the 13 instances of type 33B fracture, both observers reached a perfect DCS of 5 utilizing either imaging modality.

## Discussion

The most important findings of the present study were the superior performance of CR imaging over VR imaging in the classification of distal femur fractures and the markedly enhanced intra- and inter-observer agreement associated with the use of CR. Our results notably showed that the Kappa coefficient for CR imaging approaches perfection, indicating near impeccable reliability. In comparison to the current literature that predominantly emphasizes the utility of VR imaging, our study underscores the clinical advantage of CR imaging, which not only produces images that closely resemble actual anatomical specimens but also provides an immersive experience for orthopedic surgeons. Such realism potentially enhances the surgeon’s ability to discern intricate details of distal femur fracture injuries, consequently boosting their diagnostic confidence. While both imaging modalities demonstrated commendable accuracy, precision, recall, and F1 scores, it’s pertinent to note that CR imaging, albeit by a small margin, held an edge. The discernible difference in the DCSs further consolidates the potential of CR as a superior imaging methodology for distal femur fracture classification.

Distal femur fractures, though relatively rare, representing 0.5% of all fractures and 6% of femur fractures, are intrinsically complex with a high disability rate (Martinet et al., 2000; Pietu et al., 2014; Elsoe et al., 2018). Almost all such fractures necessitate surgical intervention, barring any conspicuous contraindications (Lim et al., 2022). Accurate preoperative diagnosis and comprehensive preoperative planning are vital and have been reported to correlate with improved clinical outcomes (Victor and Premanathan, 2013; Zeng et al., 2016; Lou et al., 2017).

From the initial dependence on X-rays, the precision of preoperative fracture classification has evolved substantially with the routine utilization of 3D CT scans (Brouwer et al., 2012). In the current investigation, orthopedic surgeons, irrespective of their years of experience, were able to perform distal femur fracture classification employing the VR technique with an accuracy rate nearing 80%. This figure was amplified to 90% upon the use of the CR technique. This upsurge was attributed to the superior imaging quality, enhanced visualization of the fracture injury details, and a presentation of the fracture that bore closer resemblance to the intraoperative view offered by CR images.

Eid et al., in a recent review, underscored the potential value of CR in trauma assessment. Preoperative treatment planning predicated on CT images is currently implemented across surgical specialties such as trauma, genitourinary, and cardiothoracic surgery and can be amplified by cine-rendered images. Eid also professed that cine-rendered 3D images permit more realistic visualization and monitoring of anatomical variations, thus offering a clearer perspective on complex issues encountered during surgery (Eid et al., 2017).

Further, in a review by Dappa et al., 2016 the potential value of CR *versus* traditional VR images was compared, with instances of potential clinical applications of CR, such as assisting in preoperative treatment planning. They emphasized that CR has superior clinical applications for structures with high density and high contrast, such as bones.

In 2017, Rowe et al. showcased CR images of musculoskeletal bones, underscoring their value in illustrating complex fractures and the relationship of fractures to adjacent soft tissues and the vascular system. They argued for the merits of CR as a valuable 3D reconstruction visualization technique that provides clinicians with a realistically rich image reading experience, albeit with a caveat that further research is needed to investigate the potential value of CR technology compared to other established reconstruction techniques (Rowe et al., 2018).

Our study echoed similar findings that CR images were more proficient in exhibiting details of comminuted intra-articular fractures compared to traditional VR imaging techniques (Figure 3). Two evaluators of varying clinical experience achieved a precision, recall, and F1 score of 1 and a 100% accuracy when employing the CR technique to AO/OTA type 33C3 fractures.

These findings suggest that CR images, based on CT thin-layer data, deliver a higher film quality and present anatomical details with greater accuracy. This is particularly beneficial for orthopedic surgeons to comprehend fracture patterns and injury specifics such as displacement of the fracture fragment, comminution of the articular surfaces, and stability of the medial and lateral columns of the distal femur. It further impacts the orthopedic surgeon’s ability to formulate precise preoperative plans, such as selecting the appropriate surgical approach and placement of internal fixation. Moreover, when sharing surgical experiences with other specialists and communicating with patients, CR images may prove invaluable, thereby promoting the evolution of orthopedic surgery and fostering harmonious relationships between doctors and patients.

This investigation has some limitations should be stated. Firstly, it is a retrospective study comprising a relatively modest cohort of only 82 cases. The low incidence of distal femoral fractures inherently limits the number of available patients, which in turn affects the sample size and diversity. This limitation might influence the statistical power and generalizability of our findings, particularly in the context of comparing CR and VR imaging modalities. Secondly, the study predominantly features non-complex fracture cases, which may not fully represent the spectrum of complexities encountered in clinical scenarios. This skew towards simpler cases limits the applicability of our findings to more complex fracture scenarios, potentially affecting the comparative analysis of the imaging techniques. Additionally, the evaluation process involved only two assessors. The variations in clinical experience between these assessors might have impacted the results. Future iterations of this research should involve a larger roster of assessors with diverse clinical experiences to validate the veracity of our conclusions. Another aspect yet to be explored is whether fracture classification via the CR technique correlates with improved clinical outcomes. To delve further into this matter, we intend to conduct a randomized controlled trial. Furthermore, as this study pioneers the comparison of CR and VR techniques in distal femur fracture classification, the absence of comparable results from other studies leaves our findings without a benchmark. This underscores the novelty of our research but also points to the need for more studies in this area. We fervently hope that our work will inspire more scholars to undertake similar studies, thereby advancing the use of CR and VR techniques in the diagnosis and treatment of fractures. The limitations of our current study, while posing challenges, also open avenues for future research to build upon our initial findings and contribute to the evolving body of knowledge in this field.

## Conclusion

Compared with traditional VR imaging, CR imaging offers a more lifelike and detailed depiction of distal femur fractures. This enhanced visualization aids orthopedic surgeons in the accurate classification of fractures, thereby enhancing their diagnostic confidence. The consistency observed in inter- and intra-observer evaluations further underscores the reliability and reproducibility of CR imaging, advocating its broader clinical application.

Looking forward, there are several promising avenues for future research in this domain. Firstly, expanding the study to include a wider variety of fracture types, particularly more complex cases, could provide deeper insights into the capabilities of CR imaging. Additionally, exploring the integration of CR imaging with other diagnostic tools and technologies, such as artificial intelligence and machine learning algorithms, could revolutionize fracture diagnosis and treatment planning. Another important area for future investigation is the assessment of the impact of CR imaging on clinical outcomes, including surgery planning, patient recovery times, and long-term prognoses.

Ultimately, as the field of medical imaging continues to advance, the potential for CR imaging to become a pivotal tool in orthopedic diagnosis and treatment is significant. Our study lays the groundwork for this advancement, and we eagerly anticipate further research that builds upon our findings to fully realize the benefits of cinematic rendering in clinical practice.

## Data Availability

The original contributions presented in the study are included in the article/Supplementary Material, further inquiries can be directed to the corresponding authors.

## Abbreviations

2D, Two-dimensional; 3D, Three-dimensional; AO/OTA, Arbeitsgemeinschaftfür Osteosynthesefragen Foundation and the Orthopaedic Trauma Association; BMI, Body mass index; CI, Confidence intervel; CR, Cinematic rendering; CT, Computed tomography; DCA, Diagnostic confidence score; DICOM, Digital Imaging and Communications in Medicine; SD, Standard deviation; VR, Volume rendering.
