# Supplementary material for: Cinematic rendering improves the AO/OTA classification of distal femur fractures compared to volume rendering: a retrospective single-center study
Source: Front Bioeng Biotechnol. 2024 Jan 8;11:1335759. doi: 10.3389/fbioe.2023.1335759 (PMC10801158; doi:10.3389/fbioe.2023.1335759)
Supplement: Supplementary file 1 [file DataSheet1.PDF]

Supplementary materials for

Cinematic Rendering Improves AO/OTA Classification of Distal Femur Fractures Compared to Volume Rendering

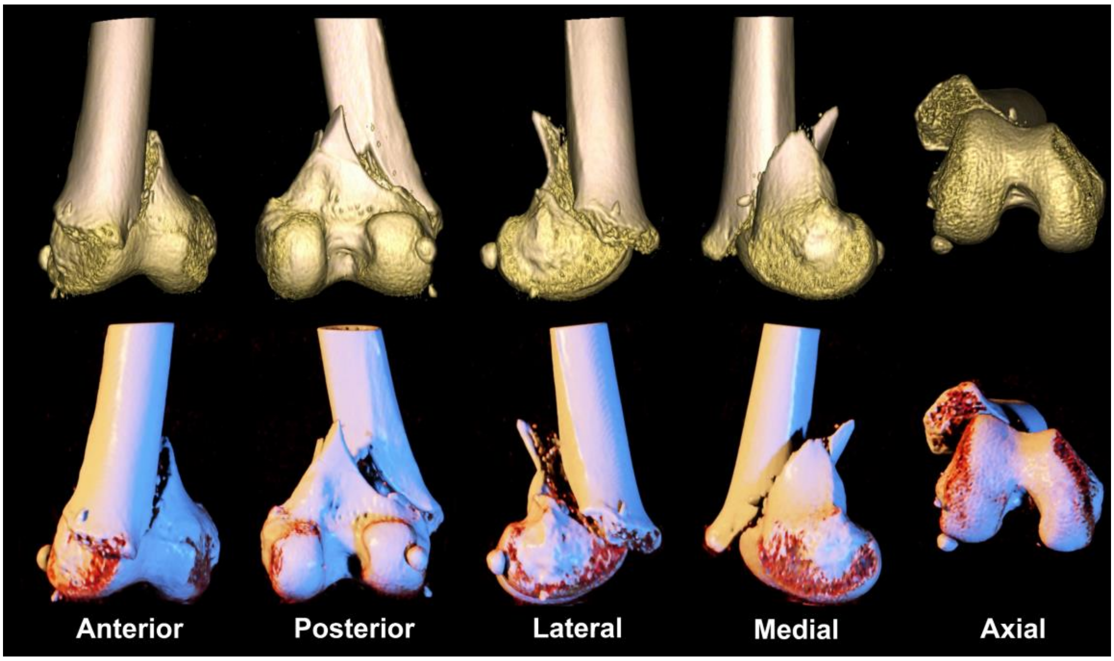

Figure S1. A 64-year-old male with a metaphyseal fracture of the right femur and a wedge-shaped bone fragment on the posterior medial side is classified as having type AO/OTA 33A2. The first row depicts a volumetric rendering reconstruction, while the second row showcases a cinematic rendering reconstruction.

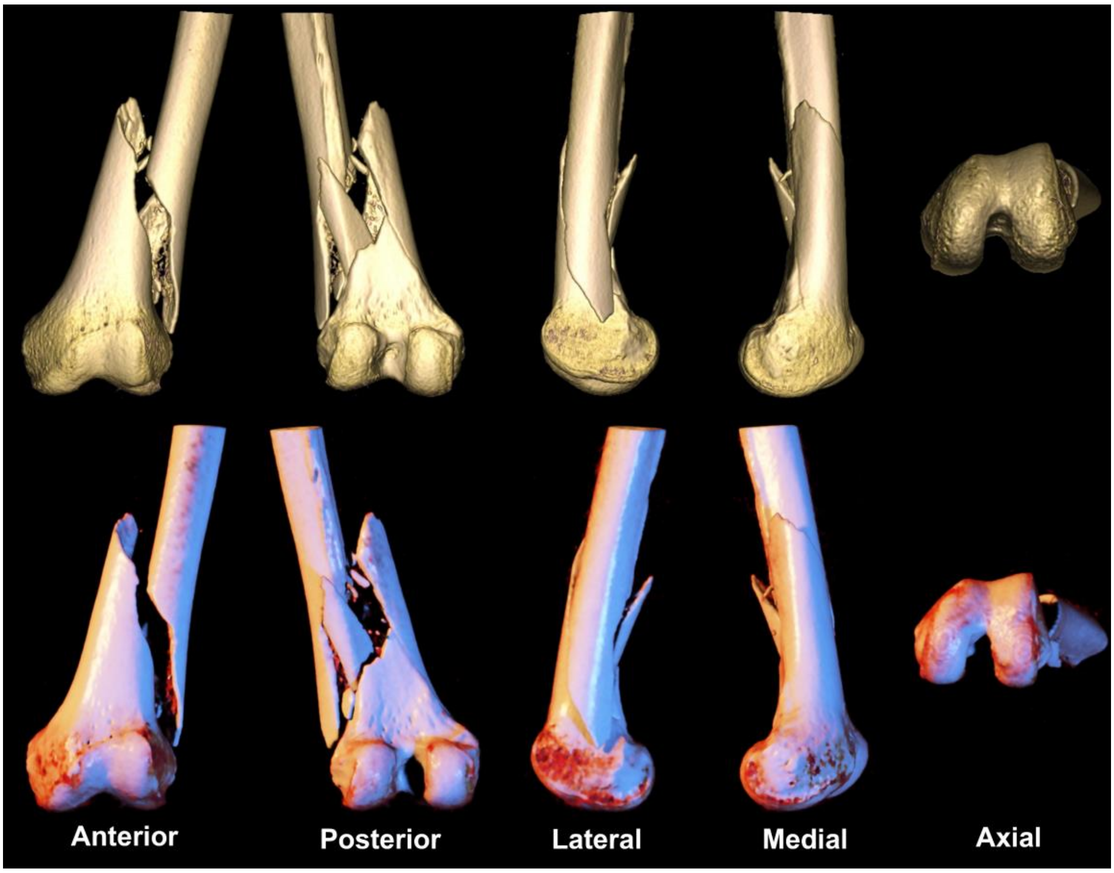

Figure S2. A 52-year-old female had a fracture on the metaphysis of the left femur; the comminuted area was concentrated on the posterior side; and the AO/OTA classification was 33A3 type. The first row depicts a volumetric rendering reconstruction, while the second row showcases a cinematic rendering reconstruction.

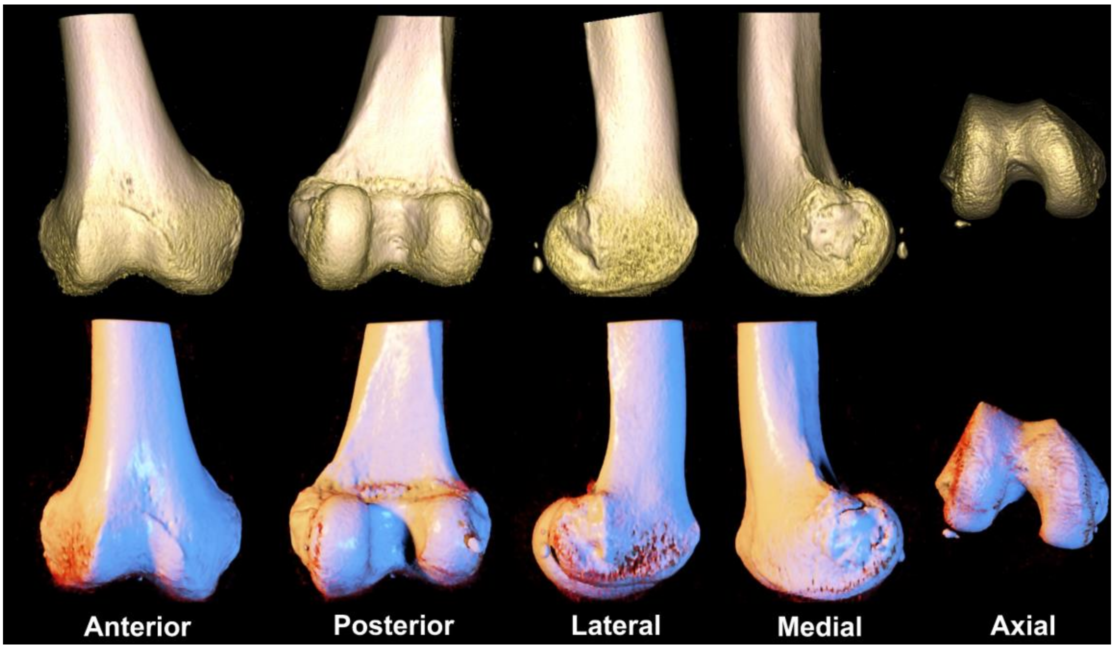

Figure S3. A 49-year-old female with a fracture on the posterior part of the right medial femoral condyle. The fracture was stable, and the displacement was not obvious. It was classified as type AO/OTA 33B2. The first row depicts a volumetric rendering reconstruction, while the second row showcases a cinematic rendering reconstruction.

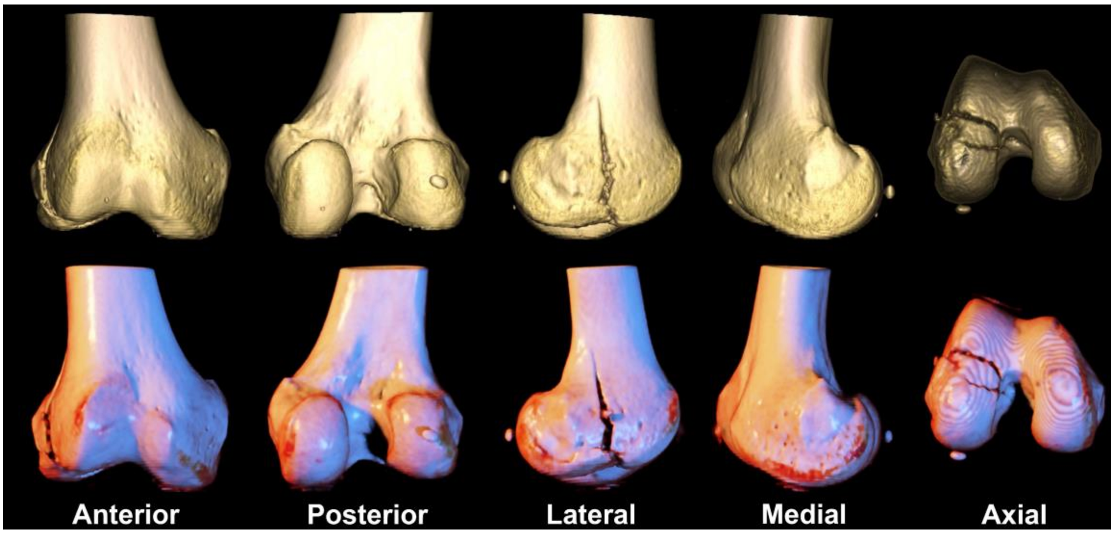

Figure S4. A 49-year-old female with a coronal fracture on the right lateral femoral condyle is classified as having type AO/OTA 33B3. The first row depicts a volumetric rendering reconstruction, while the second row showcases a cinematic rendering reconstruction.

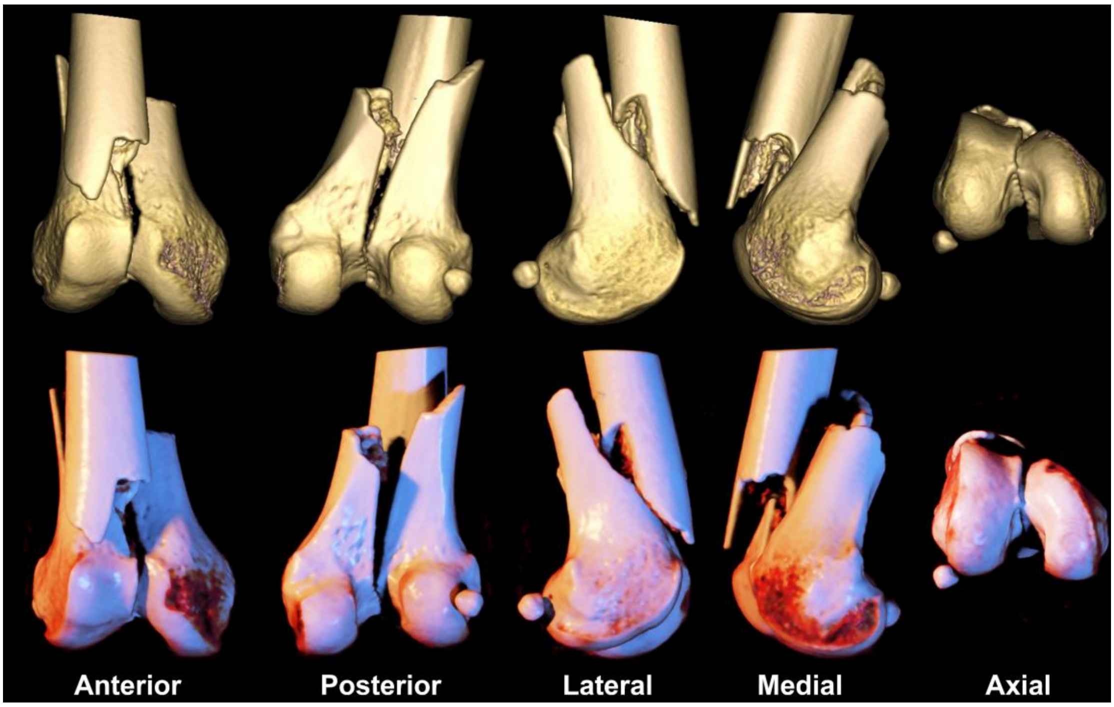

Figure S5. A 63-year-old male with a simple fracture involving the metaphysis and articular surface of the right femur is classified as having type AO/OTA 33C1. The first row depicts a volumetric rendering reconstruction, while the second row showcases a cinematic rendering reconstruction.

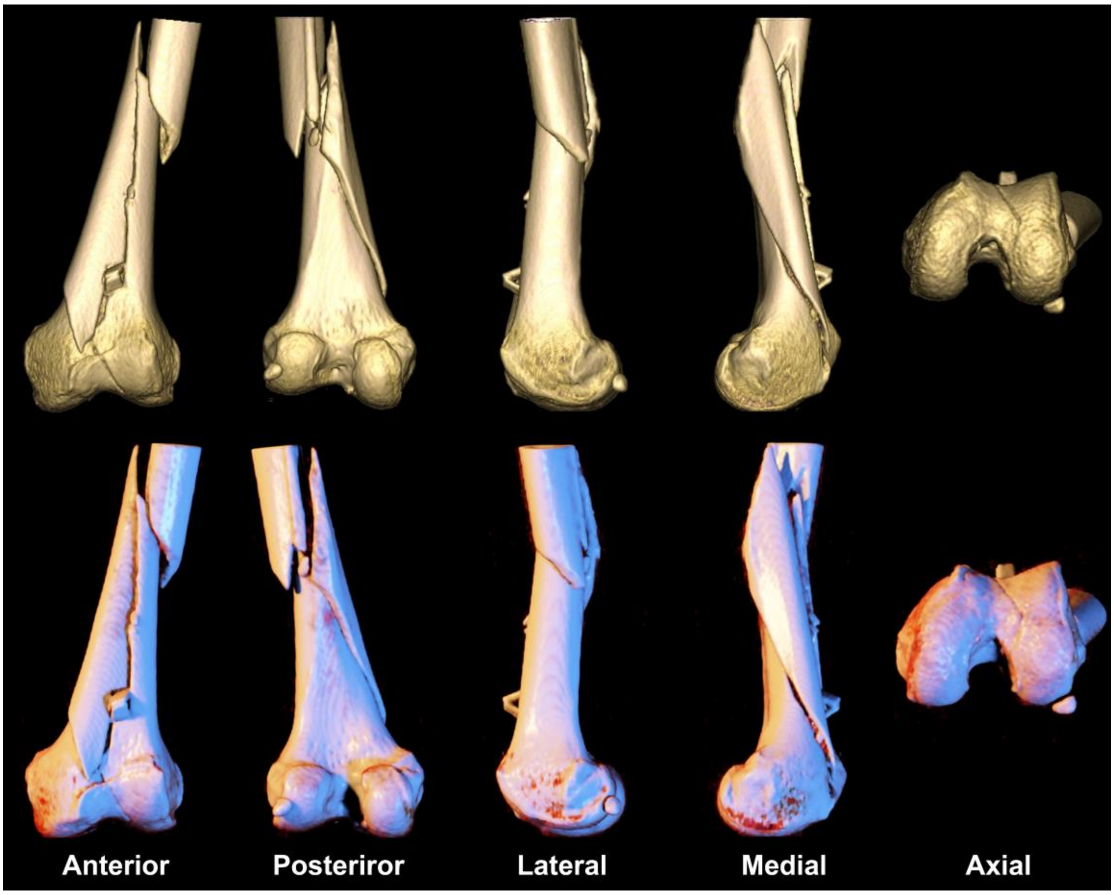

Figure S6. A 67-year-old female with a comminuted area on the left femoral metaphysis and a simple fracture of the articular surface is classified as having type AO/OTA 33C2. The first row depicts a volumetric rendering reconstruction, while the second row showcases a cinematic rendering reconstruction.
